# Culturally inclusive aging‐in‐place care preferences for persons living with dementia and their care partners

**DOI:** 10.1002/alz70863_110527

**Published:** 2025-12-23

**Authors:** Janet L. MacNeil Vroomen

**Affiliations:** ^1^ Amsterdam University Medical Center, Amsterdam, North Holland Netherlands

## Abstract

**Background:**

Aging in place (AIP) for persons with dementia is promoted, yet healthcare packages often need reassessment to align better with the preferences of persons with dementia, care partners, and care funders. Discrete choice experiments (DCEs) are valuable tools for gathering these preferences and fostering consensus among stakeholders. This Dutch national study aimed to co‐create a person‐centred healthcare package for AIP, oversampling persons with a migration background due to their higher dementia risk and under‐representation in research and care.

**Method:**

Qualitative methods (*n* = 149) identified and prioritized key care components, which were then incorporated into the DCE to quantify care needs and preferences. The study assessed both individual and joint preferences of 200 dyads using the think‐aloud method.

**Result:**

Key services included: in‐home care, assistance with household tasks and administration, social activities, emotional support, information about dementia, navigating the healthcare system, and home adaptions and tools. The study also highlighted specific care package components that are most valued by populations with migration backgrounds.

**Conclusion:**

This study provides crucial data to inform the design of person‐centred healthcare packages for AIP, ensuring that the needs of persons with dementia and their care partners are met, particularly for underserved groups such as migrants. The findings can guide the development of inclusive, culturally sensitive care models.

